# Combination of interferon-expressing oncolytic adenovirus with chemotherapy and radiation is highly synergistic in hamster model of pancreatic cancer

**DOI:** 10.18632/oncotarget.24710

**Published:** 2018-04-06

**Authors:** Amanda O. Salzwedel, Joohee Han, Christopher J. LaRocca, Ryan Shanley, Masato Yamamoto, Julia Davydova

**Affiliations:** ^1^ Department of Surgery, University of Minnesota, Minneapolis, MN 55455, USA; ^2^ Biostatistics Core, Masonic Cancer Center, University of Minnesota, Minneapolis, MN 55455, USA; ^3^ Institute of Molecular Virology, University of Minnesota, Minneapolis, MN 55455, USA; ^4^ Masonic Cancer Center, University of Minnesota, Minneapolis, MN 55455, USA

**Keywords:** oncolytic adenovirus, pancreatic cancer, interferon, combination therapy, chemoradiation

## Abstract

Recent clinical trials utilizing Interferon-alpha (IFN) in combination with chemoradiation have demonstrated significant improvements in the survival of patients with pancreatic cancer. However, efficacy was limited by the systemic toxicity of IFN and low intratumoral levels of the cytokine. We sought to address these drawbacks by using an Oncolytic Adenovirus expressing IFN (OAd-hamIFN) in combination with chemotherapy and/or radiation in regimens mimicking the IFN-based therapies used in clinical trials. IFN expressed from OAd-hamIFN potentiated the cytotoxicity of radiation and chemotherapy (5-FU, Gemcitabine, and Cisplatin), and enhanced pancreatic cancer cell death in both *in vitro* and *in vivo* experimental settings. Notably, synergism was demonstrated in therapeutic groups that combined the interferon-expressing oncolytic virus with chemotherapy and radiation. In an *in vivo* immunocompetent hamster model, treatment regimens combining oncolytic virus therapy with 5-FU and radiation demonstrated significant tumor growth inhibition and enhanced survival. This is the first study to report synergism between an IFN-expressing oncolytic adenovirus and chemoradiation-based therapies. When combined with an IFN-expressing OAd, there is a significant enhancement of radiation and especially chemoradiation, which may broaden the application of this new therapeutic approach to the pancreatic cancer patients who cannot tolerate existing chemotherapy regimens.

## INTRODUCTION

Pancreatic ductal adenocarcinoma (PDAC) is the third leading cause of cancer-related death in the United States [1]. Unfortunately, many patients are found to have advanced disease at the time of diagnosis and are not candidates for surgical resection. New chemotherapy regimens such as FOLFIRINOX [2] and NAB-paclitaxel plus Gemcitabine [3] have provided only modest improvements in survival. A lack of effective therapies against PDAC results in a five-year overall survival of 8% and has led pancreatic cancer to be projected to become the second leading cause of cancer-related death by 2017 [4].

Recently, IFN-α (IFN) based therapy regimens have appeared as a promising tool to treat pancreatic cancer. The clinical trials employing neoadjuvant and adjuvant IFN therapies are designed to combine the effectiveness of surgery with chemo-radio sensitization and the immunostimulatory effects of IFN [5–10]. Thus, clinical trials treating PDAC patients with adjuvant IFN-α (IFN) therapy in combination with radiation, 5-FU, and Cisplatin reported 16-36% increases in the 2-year survival [7, 10, 11], and a 35% increase in the 5-year survival [9, 10, 12]. Other trials included Gemcitabine in the IFN-treatment protocols and also reported a 30% increase in the 2-year overall survival of patients [11]. Despite these promising results, limitations to IFN-based therapies include dose-limiting systemic toxicities and a low intratumoral concentration of IFN due to rapid degradation of the cytokine in the blood stream [13–15].

Oncolytic viruses are a growing area of cancer research as they can be genetically modified to selectively replicate in and kill cancer cells [16, 17]. Examples of such viruses include the FDA approved T-VEC (talimogene laherparepvec) for melanoma [18] and adenovirus-based H101 therapy, which is approved in China to treat head and neck cancer [19]. Our group has previously reported the use of an Oncolytic Adenovirus (OAd) expressing human IFN-α as a promising platform for selective and long-term expression of IFN in human pancreatic cancer tissues [20, 21]. This conditionally replicative adenovirus (Ad5/Ad3-Cox2-ΔE3-ADP-IFN) was designed to selectively replicate within cancer cells expressing cyclooxygenase 2 (Cox2). To improve the infectivity and oncolysis of conventional OAds, the virus was genetically modified to include an Ad5/Ad3 chimeric fiber and overexpress the adenovirus death protein (ADP). In our later studies we have tested another OAd (OAd-hamIFN) in an immunocompetent Syrian hamster model of PDAC [22]. In contrast to mice, Syrian hamsters support human adenovirus replication [23] and provide the opportunity to analyze the immunostimulatory effect of IFN-expressing adenoviruses [23–25]. Due to a lack of Ad3 receptors on rodent cells, we have replaced the viral Ad5/Ad3 fiber with an RGD fiber [26, 27] to allow for objective evaluation of vector efficacy in a hamster model [22]. In addition, since human interferon does not bind to INF type I receptor on rodents cells [22, 28], this oncolytic virus was modified to express the hamster form of interferon [22]. OAd-hamIFN demonstrated an improved antitumor effect and extended survival when it was compared with an identical vector without IFN in both localized and disseminated PDAC hamster models [22].

Here, in the attempt to further improve effectiveness of IFN-based chemoradiation regimens, we tested the use of OAd-hamIFN in combination with 5-FU, Gemcitabine (GEM), Cisplatin (CDDP), and radiation in treatment schemes resembling the aforementioned IFN clinical trials. We hypothesized that the replication-dependent expression of IFN by an oncolytic adenovirus in PDAC tissues will potentiate the cytokine chemo- and radio-sensitization capacity and improve the therapeutic outcome of the current IFN-based regimens. To prove this hypothesis, we used the immunocompetent hamster model of PDAC as a pre-clinical platform due to the aforementioned advantages over mouse models. We hope that this work will be one more step towards translating adenovirus-based combination therapies into the clinic to improve patient outcome for this devastating disease.

## RESULTS

### Potentiation of chemotherapy by OAd-hamIFN

**Figure 1:**
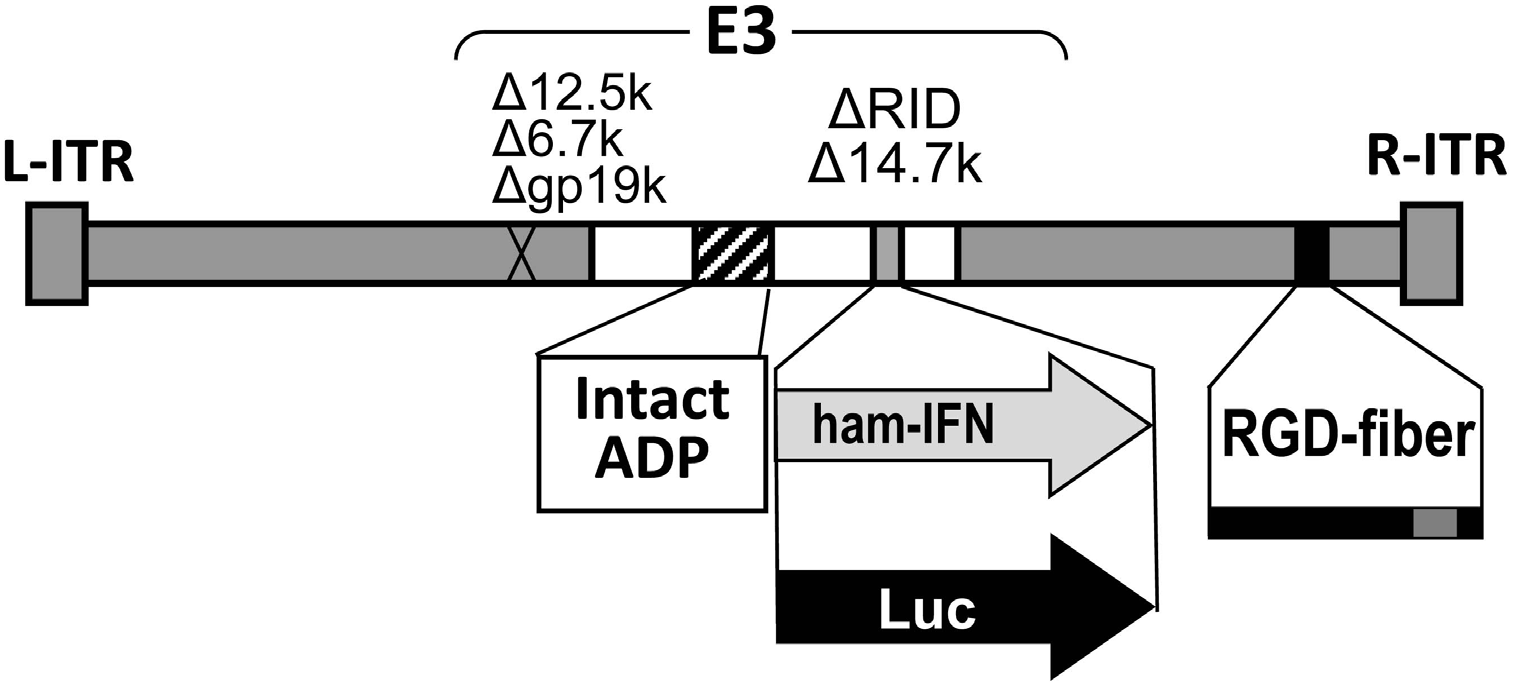
Structure of the oncolytic adenovirus expressing hamster IFN alpha (OAd-hamIFN) and the control vector expressing luciferase (OAd-Luc) OAd-hamIFN is a wild type replication oncolytic adenovirus expressing the hamster IFN-alpha gene from the adenoviral E3 region. OAd-Luc has the same structure as OAd-hamIFN, but with a luciferase transgene in place of hamster IFN.

**Figure 2:**
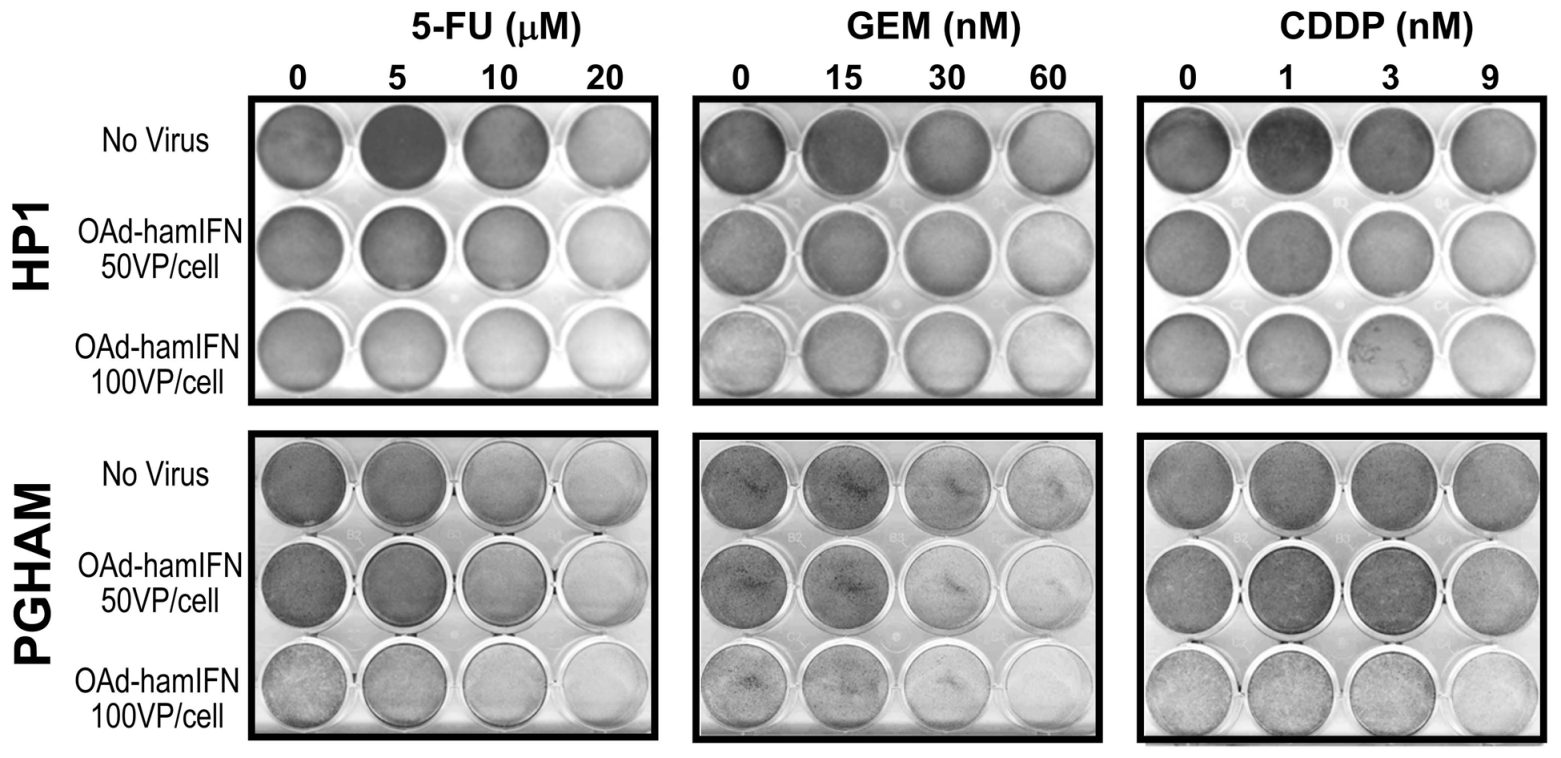
Qualitative analysis of cytocidal effect of OAd-hamIFN combined with chemotherapies Combinations of OAd-hamIFN with 5-FU, CDDP, and GEM were analyzed for the cytocidal effect by the Crystal Violet assay in HP1 and PGHAM hamster pancreatic cancer cell lines. In both cell lines, combination of virus and chemotherapy showed superior cell killing compared to cytotoxicity of virus or chemotherapy alone. The killing effect was improved with increasing of virus and chemotherapy doses. *VP: viral particle*.

**Figure 3:**
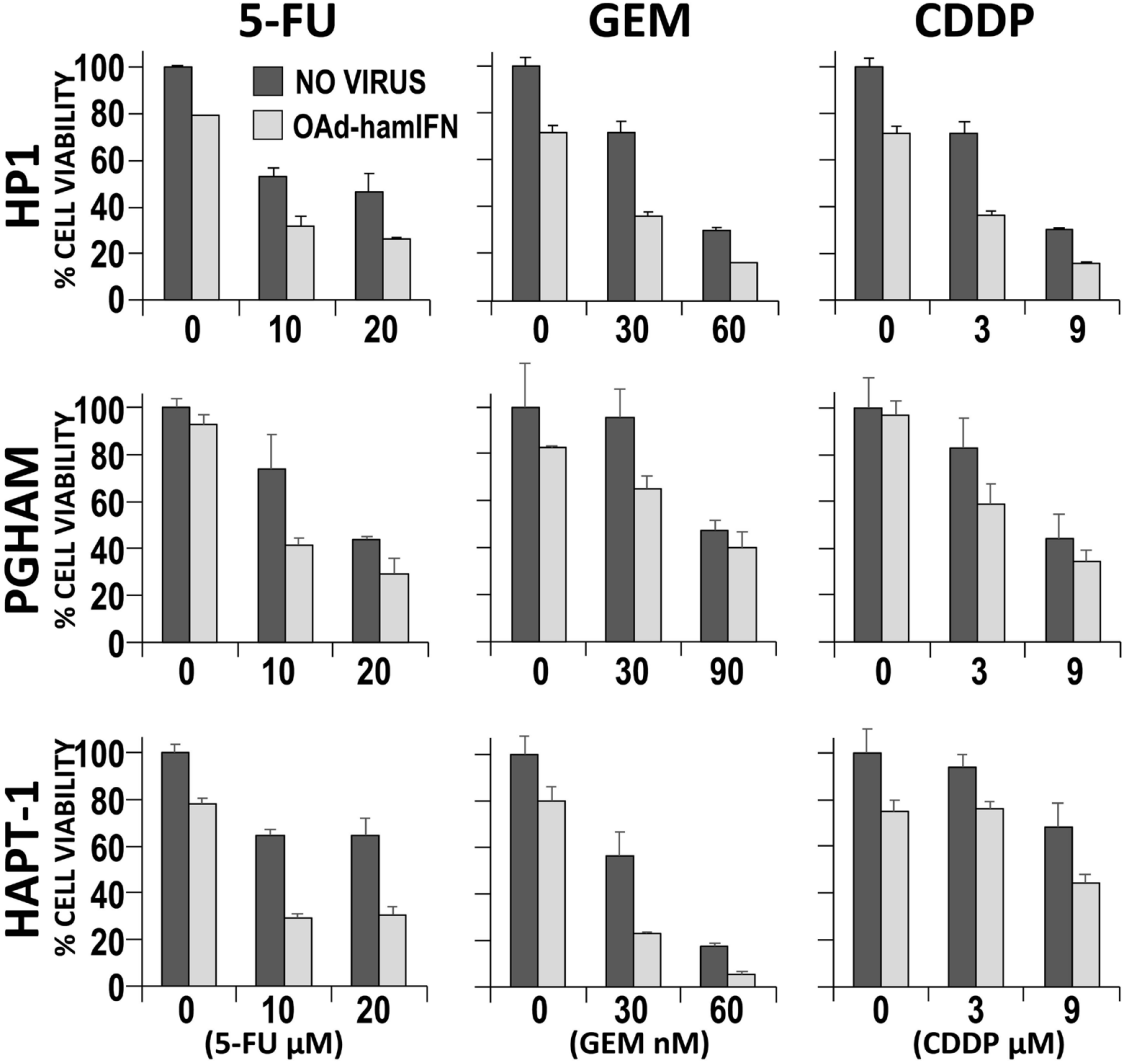
Quantitative analysis of cytocidal effect of OAd-hamIFN combination with chemotherapy Cytotoxicity of OAd-hamIFN (100 VP/cell) and chemo-monotherapies was compared to the cytocidal effect of OAd-hamIFN combination therapies with respective doses of 5-FU, GEM, and CDDP. Each column represents the mean of three experimental replicates, with the capped bars on the top of columns representing the standard deviation. Within each of the nine conditions, a two-way ANOVA was performed with dose and virus effects. All effects had *p* < 0.025, indicating that the killing effect of OAd-hamIFN+ Chemotherapy was superior to the killing effect of a drug or OAd-hamIFN alone.

The cytotoxicity of OAd-hamIFN (Figure 1) in combination with 5-FU, GEM, and CDDP was assessed *in vitro* in HP1 and PGHAM cells. The qualitative analyses by the Crystal Violet assay demonstrated the improved cytotoxic effect when 5-FU, GEM, and CDDP were combined with the IFN-expressing virus. The role of OAd-hamIFN in breaking cell resistance to chemotherapy was more evident in combination with CDDP (CDDP was not effective as a monotherapy in this setting) (Figure 2). The quantitative analyses using cell viability assays showed that compared to chemotherapy alone, OAd-hamIFN enhanced the cytotoxicity of all drugs in all cell lines tested (ANOVA *p* < 0.025 for the OAd-hamIFN effect in all nine conditions) (Figure 3). While the cytotoxicity of treatments with OAd-hamIFN and CDDP or GEM varied across the different cell lines, the use of virus with 5-FU resulted in similar cytotoxic profiles in all cell lines (29-41% mean cell viability relative to control when combined with 10 μM 5-FU, and 26-31% with 20 μM 5-FU). This data indicated that the IFN-expressing OAd can greatly accentuate the killing effect of chemotherapeutics, suggesting the possibility for dose reduction in patients.

### Contribution of IFN to chemotherapy cytotoxicity

**Figure 4:**
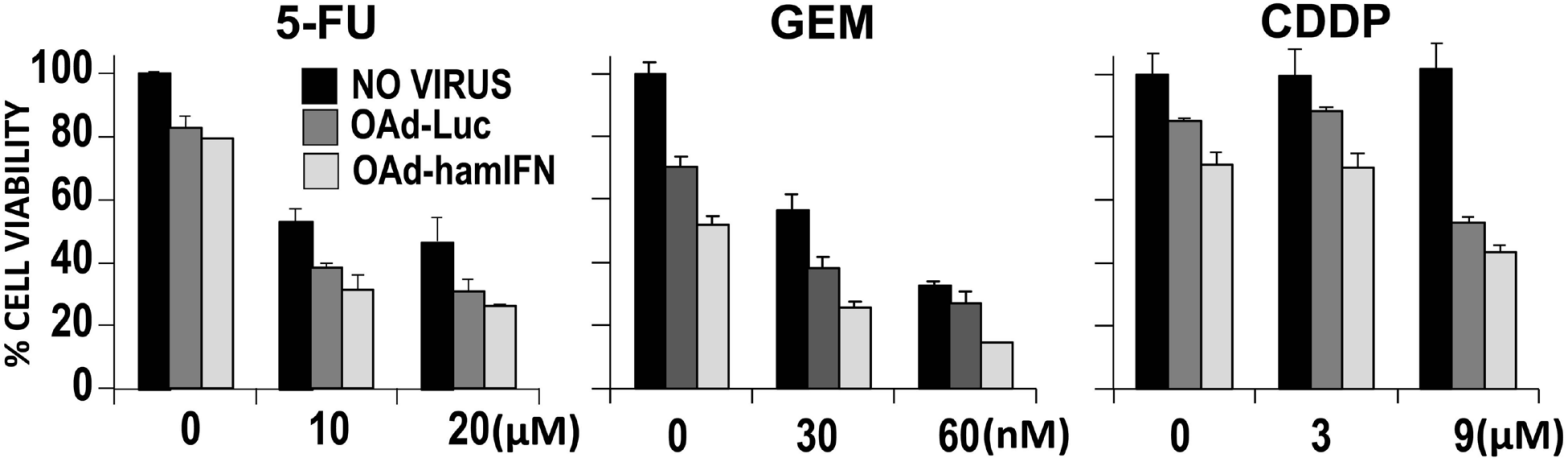
Contribution of IFN expressed by adenovirus to increase cytotoxicity of virus-chemotherapies The cell viability assay was used to evaluate contribution of IFN expression to enhance cytotoxicity of viro-chemotherapies in HP1 cells. OAd-hamIFN combined with 5-FU; GEM; and CDDP was compared to the control vector, OAd-Luc, paired with the same concentrations of chemotherapeutics. Each viral vector was used at the dose of 100 VP/cell. Each column represents the mean of three experimental replicates. The capped bars on the top of columns represent the standard deviation. Within each of the three treatment conditions, a two-way ANOVA was performed with dose and virus effects. Within each condition the contribution of IFN was significant (OAd-hamIFN versus OAd-Luc *p* -values < 0.01 for each of the three conditions).

We used a cell viability assay to analyze if IFN expressed by OAd-hamIFN can modulate the sensitivity of pancreatic cancer cells to chemotherapeutic drugs (Figure 4). To exclude the lytic effect from virus, we have employed the identical adenovirus expressing luciferase (OAd-Luc). Comparison between OAd-hamIFN and OAd-Luc showed that expression of IFN significantly improved cytotoxicity of all chemotherapeutic drugs and increased the oncolytic potential of OAd (ANOVA *p* < 0.01 for the OAd-hamIFN versus OAd-Luc effect in all three treatment conditions). For example, in the 20 μM of 5-FU condition, mean cell viability on day 7 for the no virus, OAd-Luc, and OAd-hamIFN treated groups were 46%, 31%, and 26% respectively. Importantly, expression of IFN boosted the cytotoxic effect of chemotherapy in every treatment and dose combination.

### Potentiation of radiation and chemoradiation by OAd-hamIFN

**Figure 5:**
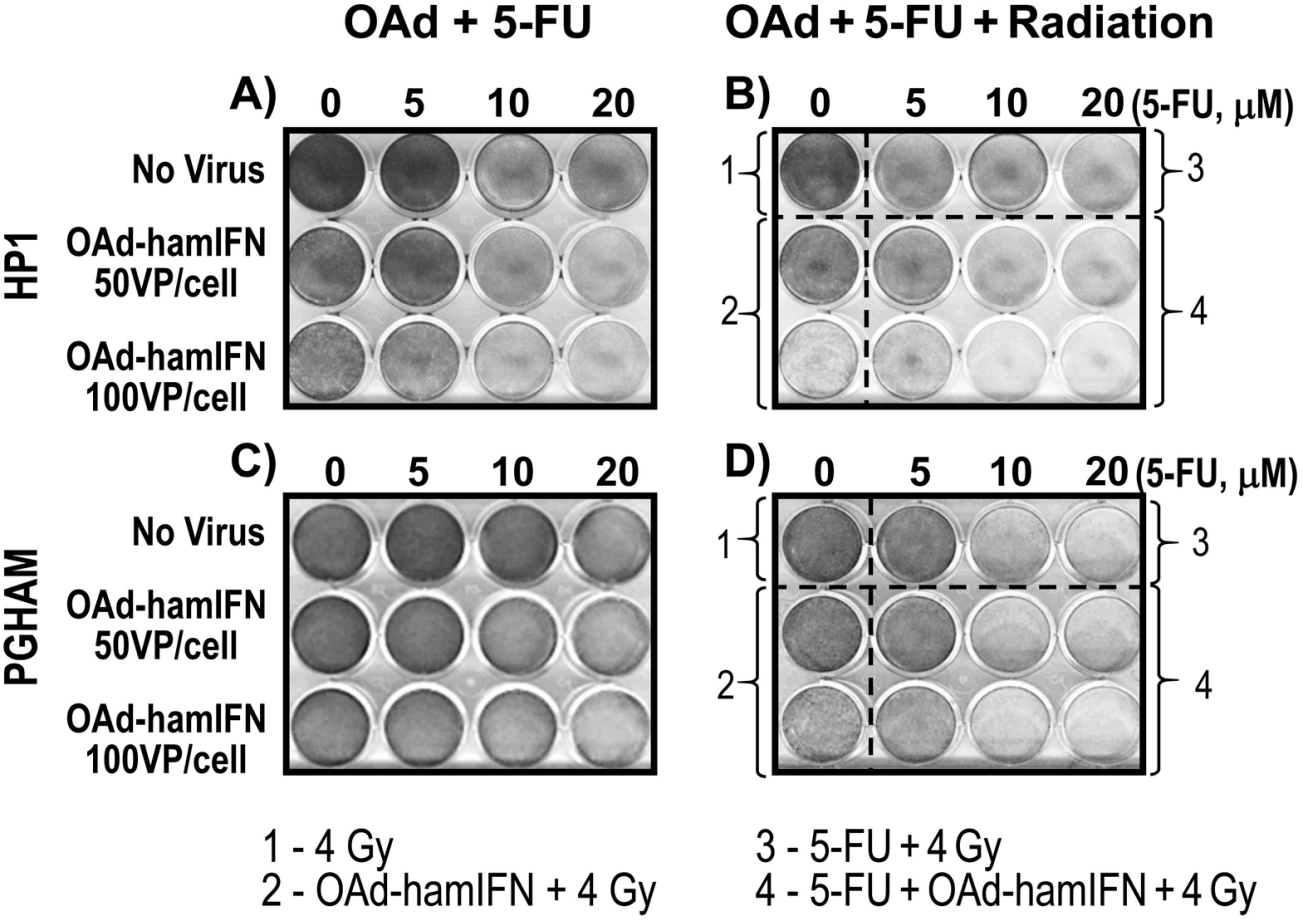
Addition of radiation to viro-chemotherapy resulted in superior killing effect Crystal Violet assay was used to assess the cytocidal effect of combination therapies with OAd-hamIFN (50 and 100 VP/cell), 5-FU (5, 10, and 20 μM), and radiation (4 Gy). The IFN-expressing OAd potentiated the killing effect of all components used in IFN-based therapies in clinical trials.

To further analyze the impact of radiation on the effectiveness of OAd-hamIFN treatments, combinations of the virus with 5-FU, radiation, and 5-FU + Radiation were tested using the Crystal Violet assay in HP1 (Figure 5A, 5B) and PGHAM cells (Figure 5C, 5D). We used 5-FU as the drug of choice because the 5-FU + radiation regimen was the standard control used to determine efficacy of IFN-based therapies in clinical trials [8–10, 29]. Qualitative assessment reiterated that OAd-hamIFN enhanced the killing effect of 5-FU (Figure 5A, 5C) and accentuated the cytotoxicity of radiation (Figure 5B-2, 5D-2). In both cell lines, the triple-therapy (OAd-hamIFN + 5-FU + Radiation) was the most cytotoxic viral combination tested (Figure 5B-4, 5D-4). Importantly, treatments including OAd-hamIFN combined with 5-FU or/and radiation, were more effective in killing cancer cells than a standard radiotherapy approach (5-FU + Radiation).

### OAd-hamIFN combinations with chemotherapy, radiation, and chemoradiation are highly synergistic

**Figure 6:**
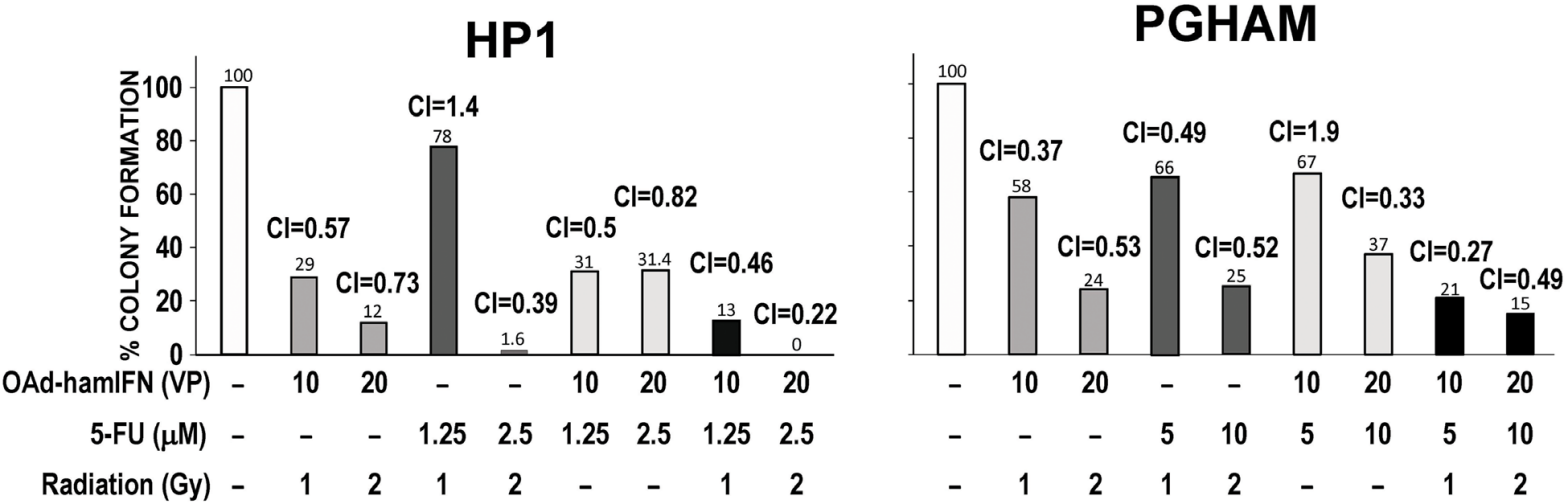
Quantification of cytotoxicity and synergy of OAd-hamIFN combination therapies based on the colony formation assay Combination of OAd-hamIFN with 5-FU, radiation, and chemoradiation reduced number of colonies formed in hamster pancreatic cancer cell lines. The strong synergism (CI < 1) was identified in groups treated with OAd-hamIFN in combination with chemotherapy, radiation, and chemoradiation. In both cell lines, combinations of OAd-hamIFN and chemoradiation in higher doses resulted in the strongest killing effect and synergistic interaction. The Chou Talalay method was used to determine the combination index (CI). Synergistic effect (CI < 1); additive effect (CI = 10); antagonistic effect (CI > 1). The numbers on the top of the histogram bars represent the percent of colonies formed after each treatment.

To characterize the interaction between the therapeutic agents combined with OAd-hamIFN, we performed a synergy analysis as described by Chou Talalay [30]. The colony formation method was employed as the gold standard assay to investigate radio-sensitivity of cancer cells (Figure 6). We have showed that treatments of OAd-hamIFN with 5-FU, radiation, and 5-FU + Radiation were efficient and inhibited formation of more than 50% of colonies post-treatment. Synergism and strong cytotoxicity was observed in all OAd-hamIFN treatments in HP1 cells. As PGHAM cells were more resistant to OAd-hamIFN, 5-FU, and radiation as monotherapies, only double-combination treatments with higher doses strongly inhibited colony formation. On the contrary, the triple-therapy regimen (OAd-hamIFN + 5-FU + Radiation) showed remarkable inhibition of colony formation regardless of the doses of chemotherapeutics. In both cell lines (PGHAM and HP1), the Combination Index (CI) analysis showed that all treatments including OAd-hamIFN were synergistic (CI < 1), and that strong synergism (CI < 0.5) was observed when OAd-hamIFN was combined with 5-FU + Radiation.

### Inhibition of tumor growth by OAd-hamIFN combination therapies in Syrian hamsters

**Figure 7:**
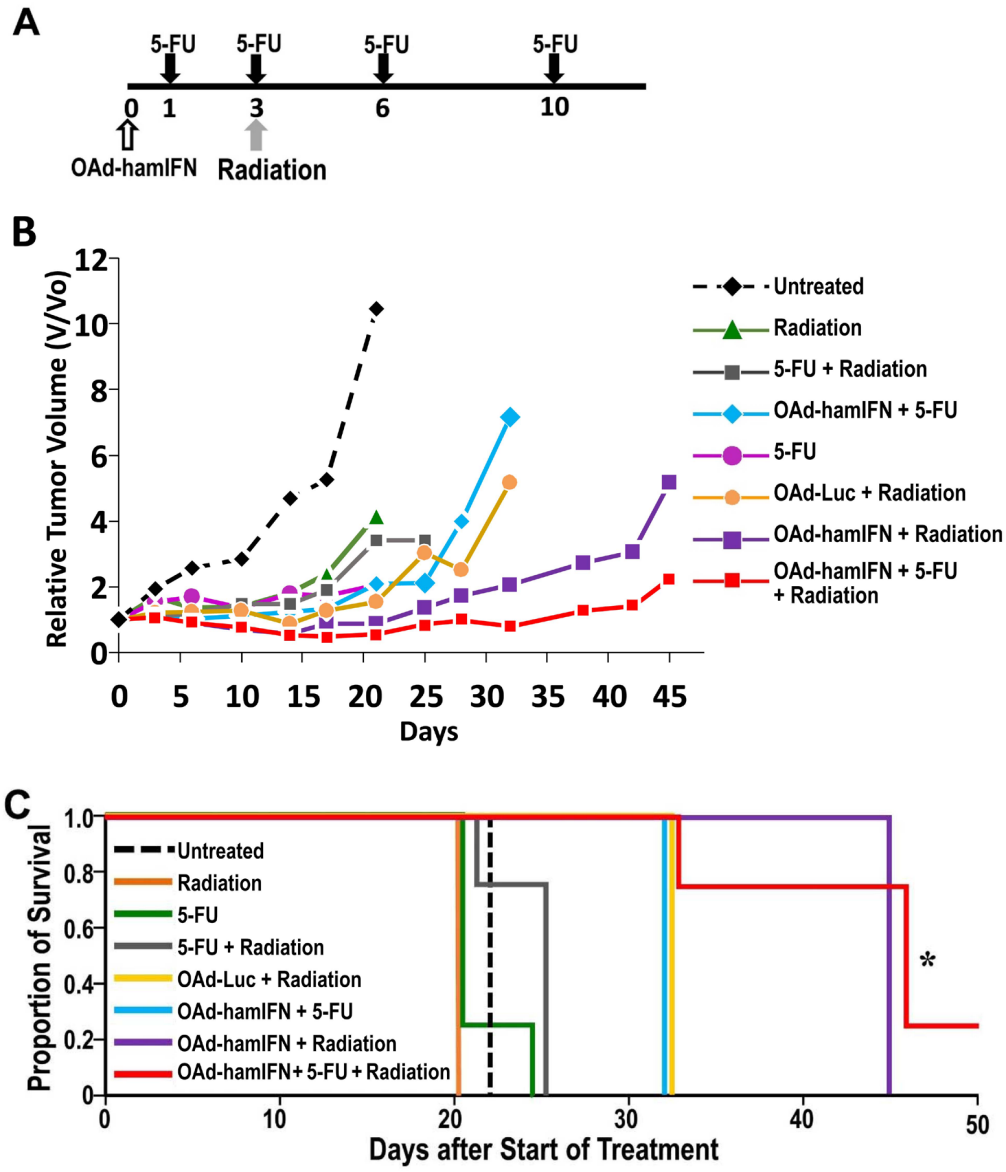
Improved anti-tumor effect and survival of OAd-hamIFN combination therapies *in vivo* PDAC hamster model **(A)** Treatment schedule in hamsters. A single fraction of radiation (8 Gy) was given 3 days after virus administration. Intraperitoneal injections of 20 mg/kg 5-FU were given on days 1, 3, 6, and 10. **(B)** Each point on the graph represents the geometric mean relative change in volume from baseline (day 0). The antitumor effect of combination therapies were compared against that of the chemoradiation group (5-FU + Radiation). The triple-therapy (OAd-hamIFN + 5-FU + Radiation) and virus-radiotherapy (OAd-hamIFN + Radiation) groups demonstrated the strongest anti-tumor effects (*p* = 0.002 and 0.01, respectively). Treatment with OAd-hamIFN + Radiation significantly outperformed treatment with OAd-Luc + Radiation (*p* = 0.02). **(C)** The survival rate of animals treated with OAd-hamIFN combined with 5FU and radiation was significantly improved when compared to that of a group treated with standard chemoradiation (5-FU + Radiation); *p* < 0.05 (^*^).

Effectiveness of OAd-hamIFN in combination with chemo- and radiotherapy was tested in the syngeneic immunocompetent hamster model of pancreatic cancer (Figure 7A). The statistical comparisons were performed against the chemoradiotherapy (5-FU + Radiation) group (conventional control used in IFN clinical trials) at day 25 post infection, before the animals in this group were euthanized due to excessive tumor volumes. All treatments including OAd-hamIFN resulted in stronger tumor growth inhibition than treatments without the virus (Figure 7B). The triple-therapy (OAd-hamIFN + 5-FU + Radiation) and virus-radiotherapy (OAd-hamIFN + Radiation) groups demonstrated the strongest anti-tumor effects (the geometric mean tumor volumes at day 25 relative to day 0 were 0.88 and 1.36, respectively, compared to 3.42 in the control group; *p* = 0.002 and 0.01, respectively). There was no significant difference between the triple-therapy group and virus-radiation group (OAd-hamIFN + Radiation) (*p* = 0.41). However, the tumor volume in the triple-therapy group was lower than baseline (day 0) until day 32 in contrast to day 21 in a group treated with OAd-hamIFN + Radiation group. Importantly, one animal treated with a triple-therapy had complete tumor elimination. The animals treated with OAd-hamIFN + 5-FU had smaller tumors than animals in the control chemoradiotherapy group; however it was not statistically significant (2.12 geometric mean relative tumor volume versus 3.42; *p* = 0.41). Statistical analysis between OAd-Luc + Radiation and OAd-hamIFN + Radiation demonstrated that IFN expressed by OAd-hamIFN significantly modulated the sensitivity of cancer cells to radiation (geometric mean relative tumor volume 3.01 versus 1.36, respectively; *p* = 0.02).

$$\text{Cellsurvival fraction}=\frac{\text{Number of colonies}}{\text{Number of plated cells}\times \text{Platingefficiency}}$$

$$\text{Plating efficiency}=\frac{\text{Number of colonies in untreated control}}{\text{Number of plated cells in untreated control}}$$

### Survival study in Syrian hamsters

Survival of hamsters treated with OAd-hamIFN combined with 5-FU, radiation, and 5-FU + Radiation was followed until 45 days after initiation of treatment (Figure 7C). Treatments with 5-FU or radiation as monotherapies were not effective, with survival similar to untreated animals (median 21 days). Animals treated with OAd-hamIFN combination treatments survived longer than monotherapy groups and the control therapy (5-FU + Radiation, median survival 25 days). Hamsters receiving OAd-hamIFN + Radiation and the triple-therapy (OAdhamIFN + 5-FU + Radiation) survived the longest, a median of 45 days (*p* = 0.07 and 0.02 versus controls, respectively). The triple therapy group was the only group to show statistically significant survival compared to the control chemoradiation group. It was also the only group to demonstrate complete tumor regression in one animal. The OAd-hamIFN + 5-FU treatment had longer survival (median 32 days) compared to the control 5-FU + Radiation group, but this was not statistically significant (*p* = 0.19). The OAd-hamIFN + Radiation group survived a median of 45 days versus 32 days in the OAd-Luc + Radiation group (*p* = 0.07), showing that expression of IFN by virus greatly contributes to improved survival.

## DISCUSSION

In this manuscript, we investigated the use of a replication competent oncolytic adenovirus expressing IFN as a tool to boost the efficacy of IFN-based chemoradiation therapy, which is one of few therapeutic protocols shown to be effective in treating PDAC.

We conducted these studies using a Syrian hamster model, as these animals are permissive to human adenovirus replication and provide a unique immunocompetent model to objectively analyze the impact of an adenovirus-produced immunostimulatory cytokine [23–25]. With this model, we assessed the use of an OAd expressing hamster IFN in therapeutic protocols similar to the IFN-based therapy used in PDAC clinical trials [5, 7, 10]. We believed that the use of an OAd, which is known to enhance the killing effect of chemotherapy and radiation [20–22, 31–34], in conjunction with a chemo-radio sensitizer such as IFN [6, 7, 10] would greatly improve the effectiveness of chemoradiation therapy.

Our data showed that IFN expressed by OAd-hamIFN augmented the capability of OAd to sensitize cells to 5-FU, GEM, and CDDP. The potentiation of the effect of these drugs is extremely important as they are commonly used to treat pancreatic cancer. We also demonstrated that the use of OAd-hamIFN with 5-FU, radiation, or 5-FU + radiation enhanced cell killing *in vitro*, and resulted in the most potent treatments *in vivo*.

The combination index (CI) analysis showed that double-therapy treatments of OAd-hamIFN with 5-FU or radiation were synergistic (CI < 1), and treatments with triple therapy (OAd-hamIFN + 5-FU + Radiation) were strongly synergistic (CI < 0.5). Based on the reports by other groups, synergistic interaction between OAd-hamIFN, chemotherapeutic drugs and radiation was expected. The oncolytic adenovirus itself can sensitize the effect of chemo- and radiotherapy [31–35]. It is well known that adenoviral E4 proteins inhibit cellular DNA repair pathways thus potentiating the effect of radiotherapy [34, 36]. Although there are no clear reports exploring the mechanism by which OAd sensitizes cells to chemotherapy drugs, the interaction between IFN and chemotherapeutics is well reported [7, 37–39]. Multiple studies report that IFN can prompt G0/G1 phase CD133+ cells to re-enter the cell cycle thus increasing therapeutic effect of nucleoside analogs such GEM. IFN induces the double stranded RNA-activated protein kinase (PKR), that shuts down protein translation [40]. It is possible that inhibition of protein synthesis combined with the DNA damage caused by 5-FU leads to synergism of OAd-hamIFN combinations with 5-FU. To exclude the lytic and sensitizing effects from the oncolytic adenovirus itself, we have employed an identical control adenovirus encoding luciferase (OAd-Luc) instead of IFN. The expression of IFN significantly enhanced the cytotoxicity of 5-FU, GEM, and CDDP in pancreatic cancer cell lines and sensitized radiotherapy in *in vivo* models, confirming the role of IFN as a chemo- and radiotherapy sensitizer.

Overall, *in vivo* data in a syngeneic immunocompetent hamster model showed that treatments with OAd-hamIFN in combination with 5-FU, radiation, and especially 5-FU + Radiation are greatly superior to that with a standard approach (5-FU + Radiation). Animals treated with the triple-combination therapy showed the strongest anti-tumor effect and survival, with one of the animals displaying complete tumor remission. There was no statistical difference between the triple-therapy group and the group treated with OAd-hamIFN + Radiation. The remarkable anti-tumor effect of OAd-hamIFN + Radiation suggests that it might be possible to reduce chemotherapy doses, broadening the application of this new therapeutic approach to the patients who cannot tolerate existing chemotherapies.

While the animals treated with OAd-hamIFN in combination with 5-FU had smaller tumors and longer survival compared to the control chemo-radiotherapy group, this was not statistically significant. The decreased efficacy of 5-FU regimens *in vivo* could be explained by the short half-life of 5-FU (approximately 30 minutes). In human clinical trials, 5-FU is continuously administered to patients to maintain constant concentration of the drug in the blood [7, 9, 11], but in our animal model drug was given with an interval of 2-4 days. While constant administration of 5-FU to animals is not feasible, it is possible that more frequent injections of the drug could have improved efficacy of OAd-hamIFN combinations with 5-FU.

Although not assessed in this project, it is possible that stimulation of anti-tumor immune response by OAd-hamIFN was also responsible for the improved therapeutic effect. The role of oncolytic viruses to initiate a robust antitumor immune response in immunocompetent models has been well reported [17, 23–25]. In fact, lytic death of cancer cells during oncolysis provides an excellent pro-inflammatory environment for induction of the antitumor vaccine response. On the other hand, IFN can stimulate maturation and activation of antigen presenting cells, activity of NK and T-helper cells, and enhances MHC-I expression [6, 9]. Supporting this hypothesis is the report by Aoki and colleagues describing that the treatment of tumor with an IFN-expressing adenovirus reduced not only the size of injected tumors, but also caused the NK-mediated anti-tumor effect in contralateral non-treated tumor [28]. Even though the tools for immunological analyses in hamsters are currently limited, further evaluation of antitumor immunity in OAd-hamIFN-treated hamsters will be necessary.

In summary, our data demonstrate the use of IFN-expressing oncolytic adenovirus in treatment protocols employing chemotherapy and radiation can provide a potent therapy for PDAC. Described synergism between an IFN-expressing OAd and chemotherapy, radiation, and chemoradiation may allow for reduction of the therapeutic doses used in IFN-based regimens, which could contribute to development of better-tolerated clinical regimens. IFN therapy is one of the few approaches found to improve the survival of pancreatic cancer patients, and the addition of an IFN-expressing OAd may leads us a step further in the development of an effective multimodal treatment against this disease.

## MATERIALS AND METHODS

### Cell lines

HP1, HAPT-1 and PGHAM hamster pancreatic cancer cells lines were provided by Dr. Hollingsworth, University of Nebraska, NE, and Dr. Uchida, Nippon Medical School, Tokyo, Japan, respectively. Cells were cultured as previously described [22].

### Adenovirus vectors

The oncolytic adenoviruses expressing hamster IFN were described previously [22]. Since the use of human Cox2 gene promoter in hamster tissues was not optimal [22] we have analyzed only the wild type replication virus in this study (OAd-hamIFN). The hamster interferon alpha gene was incorporated into the adenoviral E3 region (Figure 1). To enhance oncolysis and spreading of virus, the adenoviral death protein (ADP) was maintained in the E3 region [20–22]. To increase the infectivity of the virus in hamster pancreatic cancer cells, a RGD-4C (Arginive-Glycine-Aspartic) motif was incorporated in the HI-loop of OAd-hamIFN [26, 27]. A non-IFN expressing virus expressing firefly luciferase (OAd-Luc) was used as a control virus [41]. Virus purification, titration, and structure confirmation was performed as described previously [20, 21, 42, 43].

### Chemotherapy drugs

Fluorouracil (5-FU), Gemcitabine (GEM), and Cisplatin (CDDP) were purchased from University of Minnesota Pharmacy. Drugs were diluted and stored as described elsewhere [22]. For *in vivo* experiments, diluted 5-FU drug in the concentration of 50 mg/ml was purchased from University of Minnesota Boynton Pharmacy.

### Colony formation assay

1.0 × 10^6^ cells were seeded in 75cm^2^ flasks and incubated at 37°C in a humidified 5% CO_2_ incubator. After 24 hours, cells were infected with OAd-hamIFN or OAd-Luc. On the following day, cells were irradiated, trypsinized with 0.25% Trypsin-EDTA, counted, serially diluted, and plated in 10 cm culture dishes with standard media (DMEM with 10% FBS, 1% Penicillin/Streptomycin). Plates receiving chemotherapy as part of the treatment had 5-FU added to media at the time of plating. Media was replaced in all plates every two days. After 14 days, the plates were fixed with 4% formalin for 1 hour, stained with 1% methylene blue overnight, washed with PBS, and allowed to air dry. Colonies were counted and the following formula was used to estimate assay results:

### Cell viability assay

For quantitative analyses, 8 × 10^3^ cells/well were plated in 96-well plates, and infected with 100 viral particles (VP)/cell of OAd-hamIFN or OAd-Luc. Four hours after viral inoculation, infectious media was replaced with DMEM with 5% FBS, 1% Penicillin/Streptomycin containing 5-FU; GEM, or CDDP, and plates were incubated further. Viability assay was done with CellTiter-96®Aqueous One-Solution Cell Proliferation Assay MTS reagent (Promega) according to manufacturer's instructions.

For the Crystal Violet Assay, 2×10^5^ cells were plated in 12-wells plates and infected with 50 or 100 VP/cell. Cells were fixed and stained as described previously [22].

### *In vivo* therapeutic studies

Hamster pancreatic cancer HP1 cells (2 × 10^6^) suspended in 100 μl of PBS were subcutaneously injected into both hind legs of female Golden Syrian Hamsters (obtained from Harlan Sprague Dawley). Animals were divided in 8 groups composed of 4 animals bearing 2 tumors each. When tumor diameters reached 8 - 10 mm, they were injected with 2 × 10^11^ VP diluted in 50 μl PBS. The day of virus injection was considered as Day 0.

A clinically feasible fraction of 8 Gy was given 3 days after virus administration (as we previously showed the highest gene expression from the adenoviral E3 region in solid tumors occurs 2-6 days post infection [41]. Intraperitoneal injections of 20 mg/kg 5-FU were given on days 1, 3, 6, and 10 [7, 10, 12, 44]. Animals were anesthetized with mixture of 100 mg/kg Ketamine and 15 mg/kg Xylazine and placed in customized radiation chamber where only tumors were exposed to radiation. Tumor diameter was measured two times per week using calipers. Tumor volume was calculated considering tumor volume = (width^2^ × length)/2. The animals were euthanized according to IACUC guidelines. The animals in the untreated and chemoradiation groups were euthanized earlier due to tumor size and ascites.

### Radiation

Radiation *in vitro* and *in vivo* was performed using X-RAD 320 X-ray system (North Btanford, CT). The X-ray radiation platform was positioned at 50 cm of distance from bottom of the machine, and Filter 1 (2.0 mm Aluminum/Half Value Layer 1.0mm Cu) was used. To determine the dose for radiotherapy, the Biologically Effective Dose (BED) based on the “linear quadratic model” has been used [45]. In the clinical protocol, 3 Gy x 10 fraction (BED = 59.5) was given [45]. Expecting dose reduction benefit upon combination, we set the radiation dosage as 1/4 of the full clinical dose. BED of 8 Gy single fraction is 8 x (1+8/10) = 14.4.

### Combination index analysis

Calculation of the Combination Index (CI) to determine synergism (CI < 1), antagonism (CI > 1), or additive effect (CI = 1) between virus, chemotherapy, and radiation was performed using Compusyn software [30]. The ED50 of each treatment alone (OAd-hamIFN, 5-FU, and radiation) was quantitatively determined by the Colony Formation Assay (CFA) in HP1 and PGHAM cells, and ED50 values were entered as monotherapies in Compusyn. Quantification of cytotoxicity of combination treatments was determined by CFA in same cell lines, and killing effect was entered as the combo therapies in Compusyn. Final report with CI was generated using non-constant ratio between therapies. Strong synergism was considered when CI < 0.5 and moderate synergism when CI = 0.6 to 0.9.

### Statistical analysis

For the *in vitro* cell viability study, two-way ANOVA was performed for each cell line and treatment condition, with a three-level treatment dose effect, and a binary virus effect.

*In vivo*, tumor volume over time was analyzed using a linear mixed model. The outcome (tumor volume) was square-root transformed to improve model fit. Fixed effects included treatment group, time, and the interaction of group and time. Random intercept and time effects were included for each animal. Twelve pairwise group tests (model contrasts) compared differences in tumor volume between selected treatment groups. Reported *p*-values were obtained relative to the 5-FU+Radiation group because this was the historical control group compared to IFN-based therapies in clinical trials [8–10, 29].

For the survival study, comparisons between the 5-FU + Radiation and all other treatment groups were made using standard log-rank tests. The stepdown Bonferroni method adjusted for multiple tests in the *in vivo* and survival studies.

## References

[1] Tsvetkova EV, Asmis TR (2014). Role of neoadjuvant therapy in the management of pancreatic cancer: is the era of biomarker-directed therapy here?. Curr Oncol.

[2] Marsh RW, Talamonti MS, Katz MH, Herman JM (2015). Pancreatic cancer and FOLFIRINOX: a new era and new questions. Cancer Med.

[3] Goldstein D, El-Maraghi RH, Hammel P, Heinemann V, Kunzmann V, Sastre J, Scheithauer W, Siena S, Tabernero J, Teixeira L, Tortora G, Van Laethem JL, Young R (2015). nab-Paclitaxel plus gemcitabine for metastatic pancreatic cancer: long-term survival from a phase III trial. J Natl Cancer Inst.

[4] Siegel RL, Miller KD, Jemal A (2017). Cancer Statistics, 2017. CA Cancer J Clin.

[5] Jensen EH, Armstrong L, Lee C, Tuttle TM, Vickers SM, Sielaff T, Greeno EW (2014). Neoadjuvant interferon-based chemoradiation for borderline resectable and locally advanced pancreas cancer: a Phase II pilot study. HPB.

[6] Parmar S, Platanias LC (2003). Interferons: mechanisms of action and clinical applications. Curr Opin Oncol.

[7] Nukui Y, Picozzi VJ, Traverso LW (2000). Interferon-based adjuvant chemoradiation therapy improves survival after pancreaticoduodenectomy for pancreatic adenocarcinoma. Am J Surg.

[8] Schmidt J, Jäger D, Hoffmann K, Büchler MW, Märten A (2007). Impact of interferon-alpha in combined chemoradioimmunotherapy for pancreatic adenocarcinoma (CapRI): first data from the immunomonitoring. J Immunother.

[9] Knaebel HP, Märten A, Schmidt J, Hoffmann K, Seiler C, Lindel K, Schmitz-Winnenthal H, Fritz S, Herrmann T, Goldschmidt H, Krempien R, Mansmann U, Debus J (2005). Phase III trial of postoperative cisplatin, interferon alpha-2b, and 5-FU combined with external radiation treatment versus 5-FU alone for patients with resected pancreatic adenocarcinoma—CapRI: study protocol. BMC Cancer.

[10] Picozzi VJ, Abrams RA, Decker PA, Traverso W, O’Reilly EM, Greeno E, Martin RC, Wilfong LS, Rothenberg ML, Posner MC, Pisters PW, and American College of Surgeons Oncology Group (2011). Multicenter phase II trial of adjuvant therapy for resected pancreatic cancer using cisplatin, 5-fluorouracil, and interferon-alfa-2b-based chemoradiation: ACOSOG Trial Z05031. Ann Oncol.

[11] Linehan DC, Tan MC, Strasberg SM, Drebin JA, Hawkins WG, Picus J, Myerson RJ, Malyapa RS, Hull M, Trinkaus K, Tan BR (2008). Adjuvant interferon-based chemoradiation followed by gemcitabine for resected pancreatic adenocarcinoma: a single-institution phase II study. Ann Surg.

[12] Picozzi VJ, Kozarek RA, Traverso LW (2003). Interferon-based adjuvant chemoradiation therapy after pancreaticoduodenectomy for pancreatic adenocarcinoma. Am J Surg.

[13] Tagliaferri P, Caraglia M, Budillon A, Marra M, Vitale G, Viscomi C, Masciari S, Tassone P, Abbruzzese A, Venuta S (2005). New pharmacokinetic and pharmacodynamic tools for interferon-alpha (IFN-alpha) treatment of human cancer. Cancer Immunol Immunother.

[14] Wills RJ (1990). Clinical pharmacokinetics of interferons. Clin Pharmacokinet.

[15] Glue P, Fang JW, Rouzier-Panis R, Raffanel C, Sabo R, Gupta SK, Salfi M, Jacobs S, Hepatitis C, Intervention Therapy Group (2000). Pegylated interferon-alpha2b: pharmacokinetics, pharmacodynamics, safety, and preliminary efficacy data. Clin Pharmacol Ther.

[16] LaRocca CJ, Davydova J (2016). Oncolytic virotherapy increases the detection of microscopic metastatic disease at time of staging laparoscopy for pancreatic adenocarcinoma. EBioMedicine.

[17] Seymour LW, Fisher KD (2016). Oncolytic viruses: finally delivering. Br J Cancer.

[18] Andtbacka RH, Kaufman HL, Collichio F, Amatruda T, Senzer N, Chesney J, Delman KA, Spitler LE, Puzanov I, Agarwala SS, Milhem M, Cranmer L, Curti B (2015). Talimogene Laherparepvec improves durable response rate in patients with advanced melanoma. J Clin Oncol.

[19] Old MO, Wise-Draper T, Wright CL, Kaur B, Teknos T (2016). The current status of oncolytic viral therapy for head and neck cancer. World J Otorhinolaryngol Head Neck Surg.

[20] Armstrong L, Arrington A, Han J, Gavrikova T, Brown E, Yamamoto M, Vickers SM, Davydova J (2012). Generation of a novel, cyclooxygenase-2-targeted, interferon-expressing, conditionally replicative adenovirus for pancreatic cancer therapy. Am J Surg.

[21] Armstrong L, Davydova J, Brown E, Han J, Yamamoto M, Vickers SM (2012). Delivery of interferon alpha using a novel Cox2-controlled adenovirus for pancreatic cancer therapy. Surgery.

[22] LaRocca CJ, Han J, Gavrikova T, Armstrong L, Oliveira AR, Shanley R, Vickers SM, Yamamoto M, Davydova J (2015). Oncolytic adenovirus expressing interferon alpha in a syngeneic Syrian hamster model for the treatment of pancreatic cancer. Surgery.

[23] Thomas MA, Spencer JF, La Regina MC, Dhar D, Tollefson AE, Toth K, Wold WS (2006). Syrian hamster as a permissive immunocompetent animal model for the study of oncolytic adenovirus vectors. Cancer Res.

[24] Dhar D, Toth K, Wold WS (2014). Cycles of transient high-dose cyclophosphamide administration and intratumoral oncolytic adenovirus vector injection for long-term tumor suppression in Syrian hamsters. Cancer Gene Ther.

[25] Li X, Wang P, Li H, Du X, Liu M, Huang Q, Wang Y, Wang S (2017). The efficacy of oncolytic adenovirus is mediated by T-cell responses against virus and tumor in syrian hamster model. Clin Cancer Res.

[26] Krasnykh V, Dmitriev I, Mikheeva G, Miller CR, Belousova N, Curiel DT (1998). Characterization of an adenovirus vector containing a heterologous peptide epitope in the HI loop of the fiber knob. J Virol.

[27] Dmitriev I, Krasnykh V, Miller CR, Wang M, Kashentseva E, Mikheeva G, Belousova N, Curiel DT (1998). An adenovirus vector with genetically modified fibers demonstrates expanded tropism via utilization of a coxsackievirus and adenovirus receptor-independent cell entry mechanism. J Virol.

[28] Ohashi M, Yoshida K, Kushida M, Miura Y, Ohnami S, Ikarashi Y, Kitade Y, Yoshida T, Aoki K (2005). Adenovirus-mediated interferon alpha gene transfer induces regional direct cytotoxicity and possible systemic immunity against pancreatic cancer. Br J Cancer.

[29] Picozzi VJ, Pisters PW, Vickers SM, Strasberg SM (2008). Strength of the evidence: adjuvant therapy for resected pancreatic cancer. J Gastrointest Surg.

[30] Chou TC (2006). Theoretical basis, experimental design, and computerized simulation of synergism and antagonism in drug combination studies. Pharmacol Rev.

[31] Leitner S, Sweeney K, Oberg D, Davies D, Miranda E, Lemoine NR, Halldén G (2009). Oncolytic adenoviral mutants with E1B19K gene deletions enhance gemcitabine-induced apoptosis in pancreatic carcinoma cells and anti-tumor efficacy *in vivo*. Clin Cancer Res.

[32] Bhattacharyya M, Francis J, Eddouadi A, Lemoine NR, Halldén G (2011). An oncolytic adenovirus defective in pRb-binding (dl922-947) can efficiently eliminate pancreatic cancer cells and tumors *in vivo* in combination with 5-FU or gemcitabine. Cancer Gene Ther.

[33] Cherubini G, Kallin C, Mozetic A, Hammaren-Busch K, Müller H, Lemoine NR, Halldén G (2011). The oncolytic adenovirus AdΔΔ enhances selective cancer cell killing in combination with DNA-damaging drugs in pancreatic cancer models. Gene Ther.

[34] Hart LS, Yannone SM, Naczki C, Orlando JS, Waters SB, Akman SA, Chen DJ, Ornelles D, Koumenis C (2005). The adenovirus E4orf6 protein inhibits DNA double strand break repair and radiosensitizes human tumor cells in an E1B-55K-independent manner. J Biol Chem.

[35] O’Cathail SM, Pokrovska TD, Maughan TS, Fisher KD, Seymour LW, Hawkins MA (2017). Combining oncolytic adenovirus with radiation - a paradigm for the future of radiosensitization. Front Oncol.

[36] Boyer J, Rohleder K, Ketner G (1999). Adenovirus E4 34k and E4 11k inhibit double strand break repair and are physically associated with the cellular DNA-dependent protein kinase. Virology.

[37] Holsti LR, Mattson K, Niiranen A, Standertskiöld-Nordenstam CG, Stenman S, Sovijärvi A, Cantell K (1987). Enhancement of radiation effects by alpha interferon in the treatment of small cell carcinoma of the lung. Int J Radiat Oncol Biol Phys.

[38] Wadler S, Wersto R, Weinberg V, Thompson D, Schwartz EL (1990). Interaction of fluorouracil and interferon in human colon cancer cell lines: cytotoxic and cytokinetic effects. Cancer Res.

[39] Hayashi T, Ding Q, Kuwahata T, Maeda K, Miyazaki Y, Matsubara S, Obara T, Natsugoe S, Takao S (2012). Interferon-alpha modulates the chemosensitivity of CD133-expressing pancreatic cancer cells to gemcitabine. Cancer Sci.

[40] Williams BR (1999). PKR; a sentinel kinase for cellular stress. Oncogene.

[41] Davydova J, Gavrikova T, Brown EJ, Luo X, Curiel DT, Vickers SM, Yamamoto M (2010). *In vivo* bioimaging tracks conditionally replicative adenoviral replication and provides an early indication of viral antitumor efficacy. Cancer Sci.

[42] Davydova J, Le LP Gavrikova T, Wang M, Krasnykh V, Yamamoto M (2004). Infectivity-enhanced cyclooxygenase-2-based conditionally replicative adenoviruses for esophageal adenocarcinoma treatment. Cancer Res.

[43] Davydova J, Yamamoto M (2013). Oncolytic adenoviruses: design, generation, and experimental procedures. Curr Protoc Hum Genet.

[44] Rocha FG, Hashimoto Y, Traverso LW, Dorer R, Kozarek R, Helton WS, Picozzi VJ (2016). Interferon-based adjuvant chemoradiation for resected pancreatic head cancer: long-term follow-up of the Virginia Mason Protocol. Ann Surg.

[45] Hall EJ, Giaccia AJ (2012). Time, Dose and Fractionation in Radiotherapy. In Radiobiology for the Radiologist.

